# Sleep Disorders in Mitochondrial Diseases

**DOI:** 10.1007/s11910-021-01121-2

**Published:** 2021-05-05

**Authors:** Valerio Brunetti, Giacomo Della Marca, Serenella Servidei, Guido Primiano

**Affiliations:** 1 Fondazione Policlinico Universitario A. Gemelli IRCCS, Rome, Italy; 2 Dipartimento Universitario di Neuroscienze, Università Cattolica del Sacro Cuore, Rome, Italy

**Keywords:** Mitochondrial disease, Mitochondria, Sleep disorders, Sleep-disordered breathing, Obstructive sleep apnea, Sleep

## Abstract

**Purpose of Review:**

We aim to summarize the sleep disorders reported in patients affected by primary mitochondrial dysfunctions and describe the association with their clinical and molecular characteristics.

**Recent Findings:**

Sleep complaints are prevalent in mitochondrial disorders. Sleep-disordered breathing is the main sleep disorder reported in mitochondrial diseases. OSA and CSA are, respectively, more frequently associated with patients characterized by the prevalent involvement of the skeletal muscle and the predominant involvement of the central nervous system. Other sleep disorders, such as restless legs syndrome, have been rarely described.

**Summary:**

Sleep disorders are frequently associated with primary mitochondrial disorders, and the clinical phenotypes affect the type of sleep disturbance associated with the mitochondrial dysfunction. A polysomnographic study should be performed in every subject with this neurogenetic disorder both at diagnosis and during follow-up for the numerous adverse clinical outcomes associated with sleep disorders and the frailty of mitochondrial patients.

## Introduction

Mitochondrial medicine, a term coined by Rolf Luft in 1994 [1], is currently a recognized field in translational medical research with important clinical implications. As one of the branches of medicine in extremely rapid evolution, the role of mitochondria dysfunction in different human conditions is deeply explored in rare and common conditions, such as primary mitochondrial diseases, neurodegenerative diseases, cardiovascular diseases, aging, and cancer [2–4, 5•]. In particular regard to primary genetic mitochondrial disorders, numerous medical subspecialties have helped to further understand the pleiotropic manifestations of mitochondrial diseases (MDs), involving branches like endocrinology, cardiology, gastroenterology, and ophthalmology [6–8]. Among the commonest forms of genetic human disorders with mutations in more than 350 genes of the mitochondrial and nuclear genomes, MDs are characterized by a primary defect in oxidative phosphorylation, the main source of cellular adenosine triphosphate (ATP). With an age of onset ranging from infancy to adulthood, the patients affected by primary mitochondrial dysfunction experience a single organ involvement or more frequently a multisystem syndrome with the most energy-dependent tissues commonly affected, such as the brain and skeletal muscle [9•, 10]. The broad clinical spectrum of MDs, including specific phenotypes characterized by the predominant involvement of the skeletal muscle defined primary mitochondrial myopathies (PMM) [11••], justifies the variety of sleep disorders documented in these genetic diseases [12•] and the emerging role of mitochondrial sleep medicine.

In this review, we aim to summarize the sleep disorders reported in patients affected by primary mitochondrial dysfunctions and describe the association with clinical and molecular characteristics of mitochondrial patients. Finally, we briefly discuss the main pathophysiological mechanisms that document the key role of the mitochondria in the sleep physiology and pathology.

## Sleep Disorders in Mitochondrial Diseases: Clinical Implications

The burden of sleep disorders in MDs has been poorly documented. To date, most of the literature on this topic is mainly based on case reports or case series and few retrospective or cross-sectional studies with a significant number of patients investigated [13–16, 17••, 18, 19••, 20••, 21••]. For a detailed review of the current literature, see Table 1.

**Table 1:** Articles reporting sleep disturbances in mitochondrial diseases

Authors	Year	Study design	Patients	No Pt	Phenotype	Genotype	Sleep study	Sleep disorder
Carroll et al.	1976	Case series	P, A	4	PEO	n/a	n/a	DVR
Johnston et al.	1984	Case report	P	1	PDCD	n/a	n/a	SRH
Kotagal et al.	1985	Case report	P	1	KSS	n/a	PSG	EDS, ASS
Tatsumi et al.	1988	Case report	A	1	ME	n/a	PSG	CSA, SRH
Barohn et al.	1990	Case series	A	3	PEO	n/a	n/a	DVR
Manni et al.	1991	Cross-sectional study	A	8	PEO	n/a	PSG	CSA and REM-related hypoventilation
Suzuki et al.	1997	Case series	A	3	MIDD	m.3243A>G	n/a	DSWPD
Sembrano et al.	1997	Case report	A	1	NARP	m.8993T>G	PSG	OSA, CSA, SRH, EDS, ASS
Araki et al.	1997	Case report	P	1	LS	n/a	PSG	ASS
Guilleminault et al.	1998	Case series	A	2	PMM	n/a	ESS, PSG, MSLT	CSA, OSA , EDS
O’Brien et al.	1998	Case report	A	1	PMM	n/a	n/a	Nocturnal and daytime hypoventilation
Yasaki et al.	2001	Case series	P, A	6	LS	m.8344A>G, m.8993T>G, m.9176T>C	PSG	CSA, OSA, ASS
Osanai et al.	2001	Case report	A	1	MELAS	m.3243A>G	PSG	DVR
Sadler et al.	2002	Case report	A	1	LHON	m.11778G>A	n/a	SRH
Pincherle et al.	2006	Case report	A	1	ME	n/a	PSG	EFHM
Sanaker et al.	2007	Case report	A	1	KSS	sDel	Polygraphy	CSA, OSA, SRH
Jeyakumar et al.	2009	Retrospective chart review	P	41	n/a	n/a	n/a	OSA
Aitken et al.	2009	Case report	A	1	PEO	*POLG*	n/a	RLS
Shuk-kuen Chau et al.	2010	Case report	P	1	LS	m.8993T>G	n/a	Apnea
Vetrugno et al.	2010	Case report	A	1	LHON	m.3460G>A	PSG	EDS, CSA
Smits et al.	2012	Cross-sectional study	A	20	PEO	m.3243A>G, m.12315G>A, m.5709T>C, m.4267A>G, sDel, *POLG*	PSG, PSQI, ESS	CSA, OSA, PLMD, RLS, poor sleep quality, EDS, ASS
Tan et al.	2013	Case report	A	1	LS	n/a	n/a	OSA, SRH
Mermigkis et al.	2013	Case report	A	1	LS	n/a	PSG	OSA, EDS, increased WASO
Mosquera et al.	2014	Retrospective chart review	P, A	18	n/a	n/a	PSG	OSA,SRH, sleep-related hypoxemia, PLMD, EDS, ASS
Gorman et al.	2015	Case–control study	A	132	n/a	m.3243A>G, m.8344A>G, sDel, nuclear DNA mutations, others	ESS	EDS
Florian et al.	2015	Cross-sectional study	A	64	PEO,KSS MERRF, MELAS, others	n/a	n/a	Sleep apnea
Parikh et al.	2019	Cross-sectional study	A	48	PEO, MELAS, MIDD, LHON, LS, others	m.3243A>G, m.8344A>G, m.10466C>T, m.11778G>A, sDel, nuclear DNA mutations	ESS	EDS
Hernandez-Voth et al.	2020	Case series	A	6	PMM	*TK2*	Oximeter	Sleep-related hypoxemia, EDS
Příhodová et al.	2020	Cross-sectional study	P, A	36	LHON, DOA	m.11778G>A, m.14484T>C, m.3460G>A, *OPA1* mutations	PSG, PSQI, ESS	OSA, RWSA, poor sleep quality, EDS
Primiano et al.	2021	Cross-sectional study	A	103	PEO, MERRF, MELAS, MIDD, others	m.8344A>G, m.3243A>G, sDel, nuclear DNA mutations, others	PSG	OSA, CSA, REM-related hypoventilation

*DOA*, dominant optic atrophy; *PEO*, progressive external ophthalmoplegia; *KSS*, Kearns–Sayre syndrome; *MERRF*, myoclonic epilepsy with ragged-red fibers; *MELAS*, mitochondrial encephalopathy with lactic acidosis and stroke-like episodes; *MIDD*, maternally inherited diabetes and deafness; *PDCD*, pyruvate dehydrogenase complex deficiency; *LS*, Leigh syndrome; *LHON*, Leber hereditary optic neuropathy; *NARP*, neuropathy, ataxia, and retinitis pigmentosa; *PMM*, primary mitochondrial myopathies; *ME*, mitochondrial encephalomyopathy; *sDel*: single mtDNA deletion; *PSG*, polysomnography; *ESS*, Epworth sleepiness scale; *PSQI*, Pittsburgh sleep quality index; *RSWA*, REM sleep without atonia; *EFHM*, excessive fragmentary hypnic myoclonus; *DVR*, depressed ventilatory response; *ASS*, altered sleep structure; *DSWPD*, delayed sleep–wake phase disorder; *SRH*, sleep-related hypoventilation; *P*, pediatric patients (≤ 16 years); *A*, adult patients (≥ 16 years)

Nuclear DNA mutations include patients with *POLG*, *TWNK*, *TYMP*, *OPA1*, *RRM2B*, *SPG*7, *TK2*, *ACAD9*, *COQ8A*, *SLC25A4*, *COX10*, *MFN2*, and *NUBPL* mutations.

### Sleep-Disordered Breathing

The most frequent sleep disorders described in the context of MDs belong to the sleep-disordered breathing (SDB) group. SDB includes a constellation of disturbances classified in four major categories: obstructive sleep apnea (OSA), central sleep apnea (CSA) syndrome, sleep-related hypoxemia disorders, and sleep-related hypoventilation disorders [22]. Each of these disorders has been associated with MDs.

The physiological changes that occur during sleep make this a critical time for the process of breathing. First, the effect of gravity in the supine position determinates a reduction of the total lung capacity and a narrowing of the velopharynx [23], both contributing to the increased upper airway resistance, as well as the reduced tonic drive of pharyngeal dilator muscles [24]. Furthermore, physiological modifications observed in breathing during sleep include a reduction of respiratory rate [25], a diminished sensitivity of chemoreceptor of the respiratory center [26], and the absence of stimulus of wakefulness drive to respiration [27]. Finally, the reduction of the muscular tone involving the accessory respiratory muscles is associated with normal diaphragmatic activity in order to guarantee an adequate ventilation. This aspect is particularly important for REM sleep characterized by a complete muscular atonia and, therefore, represents the most critical sleep stage for respiration [28]. On these bases, it is expected that patients affected by neuromuscular disorders are particularly vulnerable to develop SDB [29]. OSA is the most common subtype of SDB, and it is characterized by intermittent and repetitive episodes of partial (hypopnea) or complete (apnea) obstruction of the upper airways causing falls in blood oxygen hemoglobin saturation and disruption of sleep. Daytime symptoms include excessive daytime sleepiness (EDS), fatigue, morning headache, and cognitive or mood alterations (e.g., memory loss, irritability, and depression). In our previous paper, we described a large population of adult patients affected by MDs investigated by a polysomnographic study [19••], revealing a high prevalence of OSA (35/103, 34%). Particularly, the prevalence of this specific SDB was significantly higher in the phenotypes of MDs associated with the higher grade of muscular involvement. Interestingly, the classical risk factors for OSA described in general population, such as obesity, were not significantly associated with OSA in our MD patients. Příhodová and colleagues observed a high prevalence of OSA (22%) in patients affected by Leber hereditary optic neuropathy (LHON) and dominant optic atrophy (DOA). Interestingly, the prevalence of OSA in these patients did not differ between symptomatic and asymptomatic groups [21••]. At the same time, the burden of OSA in MDs has been also revealed in pediatric populations. In particular, Jeyakumar et al. [15] observed a higher prevalence of OSA (9.8%) in pediatric patients affected by MDs when compared with that described in the general pediatric population (2%). These findings were further confirmed by Mosquera and colleagues [17••], reporting a pediatric group of patients affected by MDs by means of video-polysomnography. In their study, the authors revealed a high incidence of SDB (10/18, 56%) with a clear prevalence of OSA (6/18, 33.3%). Interestingly, the prevalence of SDB was prominent in patients with an abnormal muscular tone, while the classical risk factors of OSA, such as adenotonsillar hypertrophy, allergic rhinitis history, and post-tonsillectomy status, were not associated with SDB, suggesting that the genetic neuromuscular disease contributes to sleep respiratory disturbances. These data seem to confirm the results reported in our research paper, documenting the key role of skeletal muscle involvement in the occurrence of specific SDB [19••]. Moreover, the presence of SBD in pediatric MD population suggests that respiratory sleep disorders are an early manifestation of disease, and therefore, polysomnography should be performed as promptly as possible in these fragile patients.

Conversely, Smits et al. [20••] found a low prevalence of OSA (1/20, 5%) in a population of 20 adult patients with chronic progressive external ophthalmoplegia (PEO), with slightly increased AHI. On the other hand, in this population, CSA was the prominent SDB (4/20, 20%). A possible explanation for the discrepancy of data between the two studies is probably to be found in the different phenotypes associated with mutations in *POLG* gene. In our recent published article [19••], the patients associated with pathological variants in *POLG* presented a PMM with PEO phenotype, while Smits and colleagues investigated the presence of SDB in subjects with *POLG* mutations and ataxia neuropathy spectrum (ANS) phenotype. In light of these considerations, it is possible to hypothesize that patients with pathogenic variants in the same gene present OSA or CSA depending on a phenotype with predominant muscular or nervous system involvement. Another possible explanation of this conflicting findings is that, at least in part, some of the observed respiratory events could be classified as “pseudo-central” [30]. These events appear mainly during REM sleep characterized by a reduction of the oro-nasal flow due the diminished intercostal muscle activity and, in turn, to a reduction of the excursion of the rib cage. However, OSA has been described in association with other phenotypes of MDs, such as Leigh syndrome (LS) [17••, 31–33], Kearns–Sayre syndrome (KSS) [34], and neuropathy, ataxia, and retinitis pigmentosa (NARP) syndrome [35]. In conclusion, literature data document that OSA is a frequent sleep disorder in MDs, especially in phenotypes with predominant skeletal muscle involvement.

As aforementioned, sleep represents a state of vulnerability for patients affected by neuromuscular disorders, including MDs characterized by different degrees of involvement of the respiratory muscles. For this reason, specific subgroups of MDs have an increased risk of developing sleep-related hypoventilation/hypoxemia [34, 36]. As consequence of muscular atonia observed during REM, hypoventilation/hypoxemia usually first appears during this sleep stage [16, 19••] and, subsequently, progresses towards NREM sleep. This is mainly due to two mechanisms: the progression of the muscular weakness due to the underline disease and the decreased ventilatory drive due to a diminished sensitivity of chemoreceptor to chronic hypercarbia. In particular, the metabolic derangement, as observed in MDs, could concur to lower the chemosensitivity to hypoxia and hypercarbia. In fact, a depressed ventilatory drive response to hypoxia/hypercapnia has been described in patients affected by PEO [16, 37, 38] and mitochondrial encephalopathy with lactic acidosis and stroke-like episodes (MELAS) syndrome [39]. In particular, sleep-related hypoventilation has been associated with primary mitochondrial dysfunction with a prominent involvement of CNS [32, 34, 35, 39–42], suggesting that also a dysfunction of the brainstem respiratory center participates to the development of the sleep-related respiratory disturbance. In light of these considerations, it is not surprising that MD phenotypes with predominant CNS involvement, such as LHON “plus” [41] and LS [32], have been associated with the central hypoventilation syndrome, a peculiar life-threatening sleep disorder characterized by ineffective breathing till respiratory arrest during the sleep.

Therefore, it is possible to hypothesize that hypoventilation observed during sleep in MDs recognizes a combined etiology involving CNS dysfunction and muscular weakness [32, 43]. Moreover, sleep-related hypoventilation/hypoxemia have been described also in mitochondrial pediatric population [17••, 33], underlining that these disorders could manifest even in the early stages of the disease.

As previously mentioned, CSA has also been described in association with MDs [16, 20••, 33, 35, 42, 44]. This specific subgroup of SDB is characterized by air-flow cessation in consequence of diminished or absent respiratory effort, due to the lack of drive to breath. CSA can be associated with wakefulness hypercapnia (hypercapnic CSA) or normocapnia (non-hypercapnic CSA) [45]. Hypercapnic CSA is the result of an impaired central drive due to a dysfunction of brainstem respiratory centers (e.g., CNS diseases, specific medications) or an impaired respiratory efference (e.g., muscular disorders, motor neuron diseases). Conversely, non-hypercapnic CSA is idiopathic or frequently related to congestive heart failure. In the latter case, a peculiar pattern of crescendo/decrescendo ventilatory pattern is observed, mostly during NREM sleep, and defined as Cheyne–Stokes breathing (CSB). MDs encompass a large variety of phenotypes with different grades of muscular, neurological, and cardiac involvement. On these bases, it is reasonable to presume that they are widely associated to CSA, and therefore, the clinical suspicion of disordered breathing of central origin should be raised in those patients with genetic mitochondrial dysfunction who manifest cardiomyopathy, as observed in a LHON patient with a severe cardiac involvement [21••] or the involvement of the CNS. In fact, most of the cases described in literature of CSA in MDs are related to clinical phenotypes such as LS [33, 46], NARP [35], and KSS [34]. CSA and a diminished ventilatory response to inhaled CO2 have been described by Manni and colleagues [16] in a cohort of patients affected by PEO, without molecular characterization but defined as “ophthalmoplegia plus” for the involvement of the central and/or peripheral nervous system. Similarly, Smits et al. [20••] revealed high prevalence of CSA (4/20; 20%) in PEO patients associated with *POLG* mutations and ANS phenotype. Although the absence of genetic data in the article of Manni et al. does not allow us to draw definitive conclusions, it is possible to speculate that a combined mechanism of impaired CNS control of respiration and respiratory muscle weakness concur to determinate sleep-disordered breathing of central origin.

### Subjective Sleep Disturbances

Subjective nocturnal sleep dysfunction, evaluated by the Pittsburgh sleep quality index (PSQI), has been reported in about 75% of patients with MDs [20••] and in 70% of patients with mitochondrial optic neuropathies [21••]. Similarly, EDS, evaluated by means of Epworth sleepiness scale (ESS), has been described in adult patients affected by primary mitochondrial disorders with a prevalence ranging from 27 [14] to 33% [18] and up to 66% in pediatric population [17••]. In both studies, the prevalence of EDS appears significantly higher than in general population. EDS appears to be prevalent (4/36, 11.1%) also in mitochondrial optic neuropathies, regardless of the presence of ocular symptoms [21••]. A single study by Guilleminault and colleagues [47] objectively evaluated EDS by means of multiple sleep latency test (MSLT) in patients with neuromuscular disorders, including two patients with MDs. Both subjects presented subjective EDS (ESS > 10) objectively confirmed by a mean sleep latency of about 8 min at MSLT. Interestingly, after correcting the underlying sleep respiratory disorder, these patients presented a normalization of both subjective and objective EDS. Other experiences indicate that a treatment of an underlying sleep disorder could ameliorate daytime symptoms correlated to a chronic sleep deprivation promoted by sleep disruption [31, 35, 43]. Conversely, subjective sleep complaints were not present in a study group of patients with concomitant sleep apnea and REM-related hypoventilation [16]. Moreover, in large cross-sectional studies the prevalence of perceived sleep dysfunction [20••] and EDS [17••, 20••] is higher than the prevalence of a concomitant sleep disorder or abnormal findings on polysomnography. Similarly, in LHON and DOA [21••], no significant correlation was observed between polysomnographic parameters and poor subjective sleep quality. Therefore, it is difficult to establish whereby EDS and subjective sleep dysfunction are a reflection of underlying sleep disorder or a direct manifestation of MDs.

### Other Sleep Disorders

Other sleep disorders, in addition to those belonging to the SDB category, have been rarely described in association with MDs. Sleep-onset and maintenance insomnia have been reported in a high prevalence of patients (15/36, 41.7%) with mitochondrial optic neuropathies [21••]. Smits et al. [20••] reported a high prevalence (7/20, 35%) of restless legs syndrome (RLS) in PEO patients: two carrying *POLG* mutations, whereas the other five are associated with single mtDNA deletion or other mutations. Surprisingly, the presence of RLS was not associated to increased sleep latency neither to worst subjective sleep quality. Regarding polysomnographic findings, they found a high prevalence of periodic limb movements (PLM) in their population (mean PLM index 25.5 events/h), and in nine cases (45%), the PLM index was higher than 15 events/h which is considered the pathological cut-off [48]. PLM were more common in those patients who complained poor subjective nocturnal sleep quality, suggesting a high prevalence of periodic limb movement disorder (PLMD) in their cohort of patients affected by genetic mitochondrial dysfunction. RLS and nocturnal leg cramps seems to be prevalent (4/36, 11.1%) in patients affected by LHON and DOA, while PLM detected on PSG were present in few patients of the same cohort (2/36, 5.6%) [21••]. A case of RLS in a patient affected by PEO with a *POLG* pathological variants has been reported by Aitken et al. [49]. In this case, the patient presented an asymmetric uptake of tracer in the putamen at DaTscan, suggesting a dysfunction of the dopaminergic system, as usually observed in Parkinson disease and idiopathic RLS [50]. Interestingly, Haschka et al. [51] reported an association between RLS and the mitochondrial iron deficiency in peripheral monocytes, suggesting that mitochondrial dysfunction can concur to aggravate RLS symptoms. Increased PLM index (pediatric cut-off > 5 events/h) has been described also by Mosquera et al. [17••] in two pediatric patients, without subjective sleep movement complaints. Finally, a peculiar sleep-related movement disorder defined as excessive fragmentary hypnic myoclonus has been reported by Pincherle and colleges [52]. In this case report, the authors describe a patient with brainstem lesions on MRI, who underwent to V-PSG, presenting sub-continuous and arrhythmic myoclonic jerks occurring during both NREM and REM sleep and associated to sleep-onset insomnia, reduced sleep efficiency, and increased wake after sleep onset (WASO). Finally, a delayed sleep–wake phase disorder, a circadian rhythm sleep disorder (CRSD), has been described in a family of diabetes mellitus associated with m.3243A>G mutation [53]. Interestingly, the circadian rhythm disorder dramatically improved after the administration of coenzyme Q10, suggesting that circadian rhythm disorder can be a direct manifestation of MD, as indicated by recent evidence of a crosstalk between the mitochondria and the circadian clock [54]. Conversely, no CRSD have been observed in a large cohort of patients with mitochondrial optic neuropathies [21••], reinforcing the hypothesis that the retinohypothalamic tract, essential for light-dependent regulation of the circadian rhythm, is sufficiently preserved in these pathologies.

### Approaches to Treatment

SDB are a frequent comorbidity of MDs, and an early recognition is crucial in order to prevent further clinical deterioration of these fragile patients. Data from the literature support that mechanical ventilation is effective to improve nocturnal breathing, to restore a normal sleep architecture [35] and to ameliorate daytime symptoms [31, 47]. Particularly, in cases associated with severe central nocturnal hypoventilation [32, 41, 44, 46], treatment is lifesaving, because this condition can lead to sudden death during sleep. An early recognition and treatment could reduce the risk of further respiratory deterioration and the need for invasive mechanical ventilation or tracheotomy [32, 34, 35, 41, 46]. Moreover, a pharmacological treatment has been proposed [39, 40] to treat hypoventilation in these patients with drugs that stimulate hypoxic ventilatory response (e.g., aminophylline, theophylline, almitrine) with inconsistent results. The treatment of the underlying metabolic disorder associated with MD could also concur to ameliorate the SDB [31, 42, 43]. For pediatric population, adenotonsillectomy has been proposed for the treatment of OSA, in the absence of sufficient data to support this approach [15]. Therefore, a combined treatment with ventilatory support and pharmacological therapy appears desirable to treat SDB in MDs.

## Sleep Structure

Few data are available regarding sleep structure in patients with MDs. Sporadic and not recent articles reported the alteration of sleep macrostructure in these patients, and it seems to depend on the degree of the involvement of CNS [42, 55, 56] or on the presence and the severity of an overlapping sleep disorder [16, 44, 52]. In our previous study [19••], we observed an increase of wake after sleep onset (WASO) in the study group, whereas a reduction of slow-wave sleep (SWS) was present only in patients who suffered from SDB. These data indicated that the SDB worsen sleep quality and sleep structure in primary mitochondrial disorders. Similarly, Smits et al. [20••] observed an unstructured sleep as a consequence of SDB in a PEO population, reporting a diminished total sleep, sleep efficiency, and REM sleep in patients with pathological AHI index. The treatment of underlying sleep disorder in some cases was associated with the amelioration of sleep architecture [31, 35]. In LS [33] a decreased SWS and absence of REM stage sleep have been reported in patients who presented lower medullary lesions, regardless the presence of associated sleep disorder. In LHON and DOA, no significant alterations of sleep architecture have been observed, despite the presence of subjective sleep dysfunction, excessive daytime sleepiness, or concomitant sleep disorders [21••]. Conversely, in patients affected by mitochondrial optic neuropathies, the authors revealed the presence of REM sleep without atonia (RSWA), speculating that it is a marker of brainstem dysfunction.

To date, data regarding sleep architecture and its association with clinical phenotype and concomitant sleep disorders are inconsistent; therefore, further studies are needed to clarify this point.

## Pathophysiological Mechanisms

The mitochondrial function has a recognized pivotal role in skeletal muscle physiology for the involvement of an extraordinary number of critical processes in this high-energy-dependent tissue, such as the generation of the ATP and the regulation of energy-sensitive signaling pathways, calcium homeostasis, modulation and production of reactive oxygen species, apoptosis, and more generally cell metabolism [57]. Consequently, mitochondrial dysfunction is associated with a plethora of pathological conditions affecting the skeletal muscle, including mitochondrial myopathy [11••]. With this background, it is not surprising that patients affected by primary mitochondrial disorders characterized by the involvement of skeletal muscle (e.g., PEO and MERRF) [58, 59] presented specific subgroups of SDB, such as OSA and REM-related oxygen desaturations [19••]. Similarly, the central role of the mitochondria in numerous mechanisms of the CNS [60] may partly explain the coexistence in these patients of sleep disorders such as CSA [20••]. In addition to the data reported so far, there are no experimental models that document the direct role of mitochondria dysfunction in their genesis of sleep disorders or that explain the association with MDs. On the contrary, in recent years, a large number of research articles have documented the consequences of sleep disorders in mitochondrial functions. Rodrigues and colleagues demonstrated in a *Drosophila melanogaster* sleep-disordered model that dysregulation of homeostatic sleep regulation resulted in mitochondrial bioenergetics function, as a consequence of mitochondrial OXPHOS system dysfunction and/or of an inhibition of the mitochondrial complexes, and generation of reactive oxygen species [61]. These data are reinforced by the evidence of alterations in the antioxidant defense biomarkers in a mouse model of chronic sleep deprivation [62] and by the results reported by Trivedi and colleagues that showed oxidative stress induction and ATP depletion in patients undergoing sleep deprivation [63]. Furthermore, the imbalance between reactive oxygen species production and antioxidant defense mechanisms, resulting in an oxidative stress condition, documented in patients affected by OSA is reported in a multitude of studies, suggesting also that it may contribute to increased cardiovascular risk and neurocognitive impairment [64]. Wrede et al. have provided a further contribution in understanding the consequences of sleep disorders in the mitochondrial function. They documented that the reduction of sleep duration and sleep efficiency were associated with decreased mitochondrial DNA copy number, an indirect manifestation of mitochondrial dysfunction [65].

## Conclusions

In MDs, the involvement of organs and tissues with the highest energy demands, such as the brain and skeletal muscle, is reflected in the heterogeneity of sleep disorders associated with these metabolic diseases. There are currently few published retrospective or cross-sectional studies on this topic. Most of the knowledge regarding the association of sleep disorders and MDs is the result of case reports or case series, in which patients are poorly characterized clinically and molecularly. Nevertheless, taken together, the literature data suggest some interesting conclusions: sleep disorders are frequently associated with primary mitochondrial disorders; the clinical phenotypes affect the type of sleep disturbance associated with the mitochondrial dysfunction, as shown by the high prevalence of OSA in patients with prevalent involvement of skeletal muscle and CSA in patients in whom the central nervous system is predominantly affected; for the numerous adverse clinical outcomes associated with sleep disturbances and the frailty of mitochondrial patients, a polysomnographic study should be performed in every subject with this genetic disorder both at diagnosis and during follow-up.

Prospective and multicenter studies are needed to document the incidence of sleep disorders in primary mitochondrial disorders and their role in disease prognosis.
